# Discovery of Kasugamycin as a Potent Inhibitor of Glycoside Hydrolase Family 18 Chitinases

**DOI:** 10.3389/fmolb.2021.640356

**Published:** 2021-04-07

**Authors:** Huitang Qi, Xi Jiang, Yi Ding, Tian Liu, Qing Yang

**Affiliations:** ^1^ School of Bioengineering, Dalian University of Technology, Dalian, China; ^2^ State Key Laboratory for Biology of Plant Diseases and Insect Pests, Institute of Plant Protection, Chinese Academy of Agricultural Sciences, Beijing, China; ^3^ Guangdong Laboratory for Lingnan Modern Agriculture, Agricultural Genomics Institute at Shenzhen, Chinese Academy of Agricultural Sciences, Shenzhen, China

**Keywords:** kasugamycin, chitinase, glycoside hydrolase, inhibitor, target

## Abstract

Kasugamycin, a well-known aminoglycoside antibiotic, has been used widely in agriculture and medicine to combat microbial pathogens by binding the ribosome to inhibit translation. Here, kasugamycin was discovered to be a competitive inhibitor of glycoside hydrolase family 18 (GH18) chitinases from three different organisms (bacterium, insect and human). Results from tryptophan fluorescence spectroscopy and molecular docking revealed that kasugamycin binds to the substrate-binding clefts in a similar mode as the substrate. An electrostatic interaction between the amino group of kasugamycin and the carboxyl group of a conserved aspartate in GH18 chitinase (one of the catalytic triad residues) was found to be vital for the inhibitory activity. This paper not only reports new molecular targets of kasugamycin, but also expands our thinking about GH inhibitor design by using a scaffold unrelated to the substrate.

## Introduction

The glycoside hydrolase family 18 (GH18) chitinases (EC 3.2.1.14) are key enzymes that biodegrade chitin, which is the most abundant aminopolysaccharide in nature and composed of β-1,4-linked *N*-acetyl-D-glucosamine (GlcNAc). Chitinases are universally distributed in organisms from viruses to mammals and have diverse functions that include tissue degradation and remodeling, nutrition uptake, pathogen invasion and immune response regulation (Chen et al., 2020). For example, fungi and insects use chitinases to remodel their chitin-containing extracellular matrices (cell wall of fungi and exoskeleton of insects) (Zhu et al., 2016), whereas plants and humans, which do not contain chitin, use chitinases to degrade the chitin-containing shield of pathogens and create chitooligosaccharides (CHOS) to elicit immune responses (Kumar and Zhang, 2019).

**FIGURE 1:**
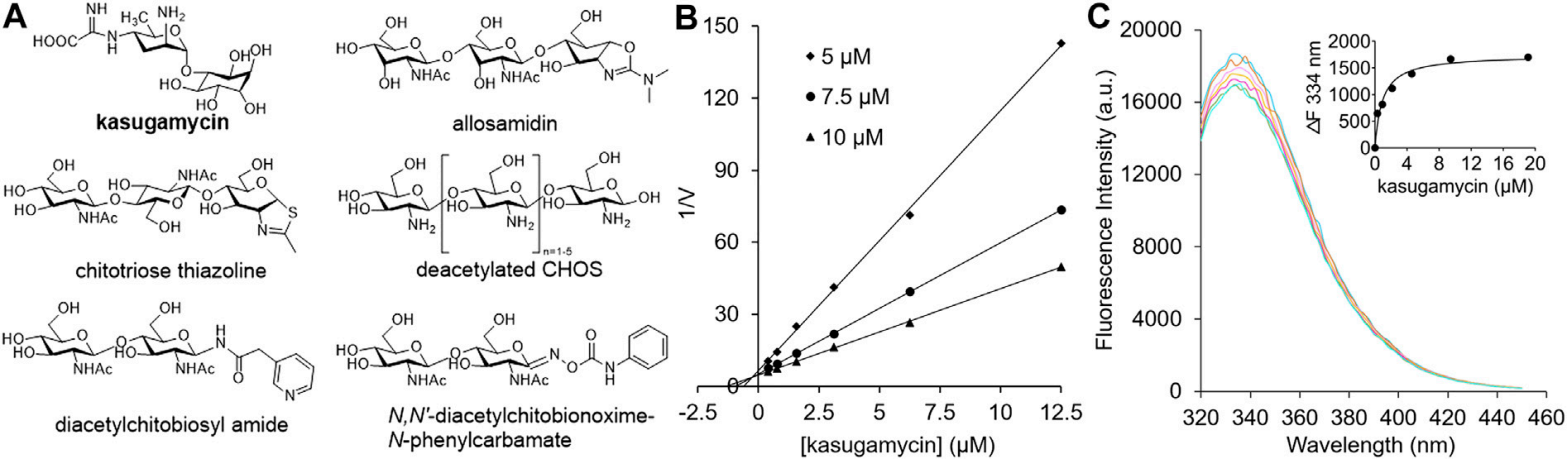
**(A)** Carbohydrate-based GH18 chitinase inhibitors. **(B)** Inhibitory kinetics of kasugamycin against *Hs*Cht. **(C)** Binding affinity of kasugamycin to *Hs*Cht.

Because GH18 chitinases play important roles in fungal pathogenesis, insect molting and human diseases, their inhibitors have potential applications as agrochemicals and human drugs (Chen et al., 2020). A core concept in the design of GH inhibitors involves simulating the structures of the substrate, transition state or reaction intermediate (Asano, 2009). Most carbohydrate-based inhibitors that target GH18 chitinases are based on these scaffolds. Allosamidin, the most studied GH18 chitinase inhibitor, is a pseudotrisaccharide composed of one *N*-acetyl-D-allosamine moiety and two GlcNAc moieties (Sakuda et al., 1986) (Figure 1A). The inhibitory mechanism involves the *N*-acetyl-D-allosamine moiety occupying the –1 subsite of the substrate-binding clefts (SBCs) and mimicking the conformation of the reaction intermediate (Pantoom et al., 2011). Chitotriose thiazolines are designed GH18 chitinase inhibitors with similar structures and inhibitory mechanisms as allosamidin (Macdonald et al., 2010) (Figure 1A). The only structural difference is the *N*-acetyl-D-allosamine moiety in allosamidin is replaced by a GlcNAc-thiazoline. In addition to reaction intermediate mimics, various substrate mimics such as deacetylated CHOS (Chen et al., 2014) (Figure 1A), diacetylchitobiosyl amides (Rottmann et al., 1999) and *N,N′*-diacetylchitoxime-*N*-phenylcarbamate (Vaaje-Kolstad et al., 2004) have been discovered as GH18 chitinase inhibitors.

Kasugamycin is an aminoglycoside agrochemical that has been used widely to treat plant bacterial and fungal diseases. Kasugamycin kills the target microorganisms by interfering with the interaction between mRNA and the 30S subunit of the ribosome (Schluenzen et al., 2006). In this study, kasugamycin was discovered for the first time to inhibit bacterial, insect and human GH18 chitinases. However, the structure of kasugamycin is totally different from that of CHOS. Furthermore, tryptophan fluorescence spectroscopy and molecular docking were used to study the mechanisms of potent inhibition as well as the relative selectivity among GH18 chitinases.

## Materials and Methods

### Materials

4-Methylumbelliferyl-β-D-*N,N′*-diacetylchitobiose [MU-β-(GlcNAc)_2_] was purchased from Sigma- Aldrich (Shanghai, China). Kasugamycin was purchased from MedChemExpress (Newark, NJ, United States). The chromatographic columns for proteins purification were purchased from GE Healthcare (Boston, United States). The SDS-PAGE Gel Kit was purchased from Solarbio (Beijing, China).

### Enzyme Preparation

The recombinant GH18 chitinases for inhibitory activity evaluation including *Of*Chi-h and the catalytic domains of *Of*ChtI from *Ostrinia furnacalis* (*O. furnacalis*), human *Hs*Cht and acidic mammalian chitinase (AMCase) were extracellularly expressed in *Pichia pastoris* GS115. *Sm*ChiA from *Serratia marcescens* was expressed in *Escherichia coli* BL21 (DE3). All the proteins were purified from the culture medium by immobilized metal affinity chromatography (IMAC) as described previously (Chen et al., 2014). The purities of the target proteins were analyzed by SDS-PAGE followed by Coomassie Brilliant Blue R-250 staining.

### Inhibitory Activity Determination

Briefly, the reaction mixtures used for inhibitor screening had a final assay volume of 100 μL. 20 nM enzyme was incubated with 10 μL substrate [0.2 μM MU-β-(GlcNAc)_2_] in 20 mM sodium phosphate buffer (pH 6.0 for *Of*Chi-h, *Of*ChtI, *Hs*Cht and *Sm*ChiA, and pH 5.2 for AMCase) containing 2 μL inhibitor at 30°C. The reaction in the absence of inhibitor and substrate was used as the control. After 30 min, an equal volume of 0.5 M Na_2_CO_3_ was added to the reaction mixture to stop the reaction, and the fluorescence of the released MU was quantitated using an Infinite® 200 PRO microplate reader (Tecan, Swiss) with excitation and emission wavelengths of 360 and 450 nm, respectively. Experiments were performed in triplicate. For the determination of the mode of inhibition and the inhibition constant (*K*_i_) value, the reaction mixtures contained three MU-β-(GlcNAc)_2_ concentrations (5, 10 and 20 μM for *Of*Chi-h, AMCase and *Sm*ChiA; 5, 7.5 and 10 μM for *Hs*Cht; and 1, 2 and 4 μM for *Of*ChtI). The inhibitor concentration varied according to the inhibitory activity. The *K*i values and types of inhibition were determined by linear fitting of data in Dixon plots.

### Tryptophan Fluorescence Spectroscopy

All tryptophan fluorescence spectroscopy experiments were performed using the Infinite® 200 PRO microplate reader (Tecan, Swiss). The excitation wavelength was fixed to 285 nm and emission spectra were collected between 320 and 450 nm with a slit width of 2 nm. The temperature was maintained at 30°C. Fluorescence quenching experiments were performed in a 100 μL mixture containing 1 μM protein in the 20 mM sodium phosphate buffer (pH 6.0 for *Of*Chi-h, *Of*ChtI, *Hs*Cht and *Sm*ChiA, and pH 5.2 for AMCase), and by the successive addition of 1 μL compounds stock solution. The inhibitor concentration varied according to the enzyme activity. The dissociation constant (*K*_d_) value was analyzed by nonlinear regression with the “One Site-Specific Binding” model using GraphPad Prism 7.04.

### Molecular Docking

The PRODRG2 server was used to generate and optimize the initial structure of the compounds before docking (Schuttelkopf and Van Aalten, 2004). Polar hydrogen atoms and Gasteiger charges were added using MGLTools. The center of the grid box was placed at the center of the active pocket of *Of*Chi-h (PDB ID: 5GQB), *Of*ChtI (PDB ID: 3WQW), AMCase (PDB ID: 2YBT), *Hs*Cht (PDB ID: 1HKK) and *Sm*ChiA (PDB ID: 1CTN). Polar hydrogen atoms and Gasteiger charges were added to proteins using MGLTools. Affinity grids of 40 × 50 × 40 Å^3^ for *Of*Chi-h, 42 × 48 × 40 Å^3^ for *Of*ChtI, 38 × 50 × 35 Å^3^ for AMCase, 40 × 40 × 40 Å^3^ for *Hs*Cht and 40 × 28 × 38 Å^3^ for *Sm*ChiA were calculated using AutoGrid4. Molecular dockings were performed by AutoDock4 using the Lamarckian genetic algorithm with a population size of random individuals, 25,000,000 energy evaluations and 27,000 generations. Plausible docking models were selected from the most abundant clusters that had the lowest binding energies. All structures were analyzed using PyMOL.

## Results and Discussion

In our preliminary screening of a natural products library, kasugamycin was found to display 92.54% inhibition against *Of*ChtI, an insect GH18 chitinase from *O. furnacalis*, at 10 μM. In this research, the inhibition activities of kasugamycin against various GH18 chitinases including *Sm*ChiA from *S. marcescens* (Fuchs et al., 1986), *Of*ChtI and *Of*Chi-h from *O. furnacalis* (Liu et al., 2017), and *Hs*Cht and AMCase from *Homo sapiens* (Fusetti et al., 2002; Bussink et al., 2008) were studied. Inhibition kinetics demonstrated that kasugamycin inhibits all of the tested GH18 chitinases in a competitive mode (Figure 1B; Supplementary Figure S1) with *K*_i_ values varying from 0.25 to 29.00 μM (Table 1).

**TABLE 1:** Inhibitory activities and binding affinities of the compounds toward different GH18 chitinases.

Organism	Name	*K*_i_ (μM)	*K*_d_ (μM)
Human	*Hs*Cht	0.25 (1.62)^a^	0.92
	AMCase	6.27	15.84
Insect	*Of*ChtI	0.47	3.96
	*Of*Chi-h	2.7	11.5
Bacterium	*Sm*ChiA	29.00	34.11

^a^ The *K*_i_ of kasugamycin against *Hs*Cht in the buffer with 1.0 M NaCl.

Since the SBCs of GH18 chitinases usually contain several solvent-exposed tryptophan residues, tryptophan fluorescence quenching spectroscopy was used to determine the binding affinity of kasugamycin to GH18 chitinases. As shown in Figure 1C and Supplementary Figure S2, kasugamycin quenched the native tryptophan fluorescence of GH18 chitinases in a dose-dependent mode. The equilibrium dissociation constant (*K*_d_) values of kasugamycin to GH18 chitinases varied from 0.92 to 34.11 μM (Table 1). The tendency of the *K*_d_ values is in good accordance with that of the *K*_i_ values, although the values are not identical.

**FIGURE 2:**
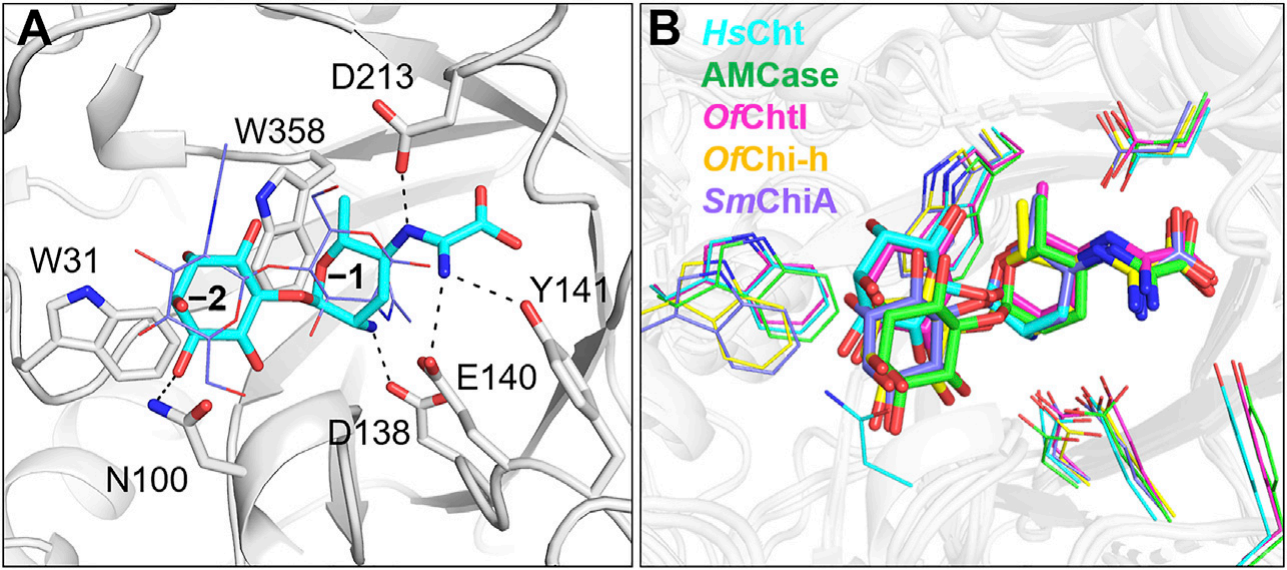
Modeled structures of kasugamycin in complex with GH18 chitinases. **(A)** Modeled binding mode of kasugamycin to *Hs*Cht. **(B)** Superposition of the modeled conformations of kasugamycin in different GH18 chitinases. Cyan in *Hs*Cht, green in AMCase, pink in *Of*ChtI, yellow in *Of*Chi-h and purple in *Sm*ChiA.

To further understand the inhibitory mechanism, kasugamycin was docked into the crystal structure of *Hs*Cht (Fusetti et al., 2002), which has the highest affinity toward kasugamycin. Although there is little structural similarity between kasugamycin and CHOS, kasugamycin bound the SBC of *Hs*Cht in a similar mode as (GlcNAc)_2_ by forming CH-π interactions with the indole group of Trp^31^ and Trp^358^ (Figure 2A). The methylkasugaminide moiety occupied the –1 subsite of the SBC and formed hydrogen bonds with surrounding residues including Glu^140^, Tyr^141^ and Asp^213^. The D-inositol moiety of kasugamycin occupied the –2 subsite of the SBC and formed a hydrogen bond with Asn^100^. Since the amino group of kasugamycin and the carboxyl group of Asp^138^ (one of the catalytic triad residues) have opposite charges at pH 6.0, we hypothesized that the strong electrostatic interaction between them was a driving force for the inhibitory activity of kasugamycin against GH18 chitinases. To prove this hypothesis, we determined the *K*_i_ value of kasugamycin against *Hs*Cht in a buffer containing 1.0 M NaCl to weaken the electrostatic interaction. Under these conditions, the *K*_i_ value of kasugamycin against *Hs*Cht increased 6-fold to 1.62 μM (Table 1; Supplementary Figure S3), demonstrating the importance of this electrostatic interaction in the binding affinity of kasugamycin to GH18 chitinases. Most of residues involved in binding were key residues for chitinase catalysis. Residues Asp^138^ and Glu^140^ are responsible for glycosidic bond breaking. Residue Asp^213^ is involved in catalysis by stabilizing the −1 sugar in its distorted conformation (Synstad et al., 2004; Chen et al., 2020). Mutation of these residues in *Sm*ChiB yielded greatly reduction in catalytic activity (Synstad et al., 2004). Kasugamycin was first reported as a bacterial protein synthesis inhibitor, and the binding mechanism of kasugamycin to the 30S subunit of the bacterial ribosome has been studied by X-ray crystallography (Schluenzen et al., 2006). In this structure, kasugamycin binds the 16S ribosomal RNA within the messenger RNA channel. The electrostatic interaction formed between the amino group of kasugamycin and the backbone phosphate group of G1483 was also important for defining the binding affinity.

Kasugamycin was also docked into the crystal structures of *Of*ChtI, *Of*Chi-h, AMCase and *Sm*ChiA. The modeled complex-structures with the highest frequency were then superimposed to determine the molecular basis for the relative selectivity among different GH18 chitinases (Figure 2B). The binding mode of the methylkasugaminide moiety was almost the same among the different GH18 chitinases whereas the binding mode of the D-inositol moiety differed. In *Of*Chi-h and *Sm*ChiA, Tyr^141^ in *Hs*Cht was replaced by a phenylalanine residue, which cannot participate in hydrogen bond formation (Figure 2B; Supplementary Figure S4). The D-inositol moiety occupies the –2 subsite of the SBC of *Of*ChtI but does not form a hydrogen bond with any residues (a hydrogen bond with Asn^100^ was found in *Hs*Cht). As for *Of*Chi-h, AMCase and *Sm*ChiA, which have much lower binding affinities to kasugamycin, the D-inositol moiety rotates about 28° away from the conserved tryptophan at the –2 subsite (Trp^31^ in *Hs*Cht), which may result in the loss of the CH-π interaction with this residue (Figure 2B; Supplementary Figure S4). These results indicate that the inhibitory activity of kasugamycin against different GH18 chitinases can be optimized by modifying the D-inositol moiety to increase the number of intermolecular interactions with residues surrounding the –2 subsite of the SBC.

After this article was accepted, we noticed that kasugamycin was reported by another research group as *Hs*Cht inhibitor, and its potential in the treatment of pulmonary fibrosis was also investigated (Lee et al., 2021).

## Conclusion

In this work, kasugamycin was discovered as a competitive inhibitor of various GH18 chitinases. By tryptophan fluorescence spectroscopy and structure-based molecular docking, kasugamycin was revealed to bind SBCs of GH18 chitinases in a similar mode as the substrate through an electrostatic interaction, CH-π interactions and hydrogen bonds. This work does not reveal novel targets for kasugamycin but provides a new scaffold for designing novel GH inhibitors.

## Data Availability

The raw data supporting the conclusion of this article will be made available by the authors, without undue reservation.
