# Supplementary material for: Discovery of Kasugamycin as a Potent Inhibitor of Glycoside Hydrolase Family 18 Chitinases
Source: Front Mol Biosci. 2021 Apr 7;8:640356. doi: 10.3389/fmolb.2021.640356 (PMC8058351; doi:10.3389/fmolb.2021.640356)
Supplement: Supplementary file 1 [file datasheet1.pdf]

Supplementary Material

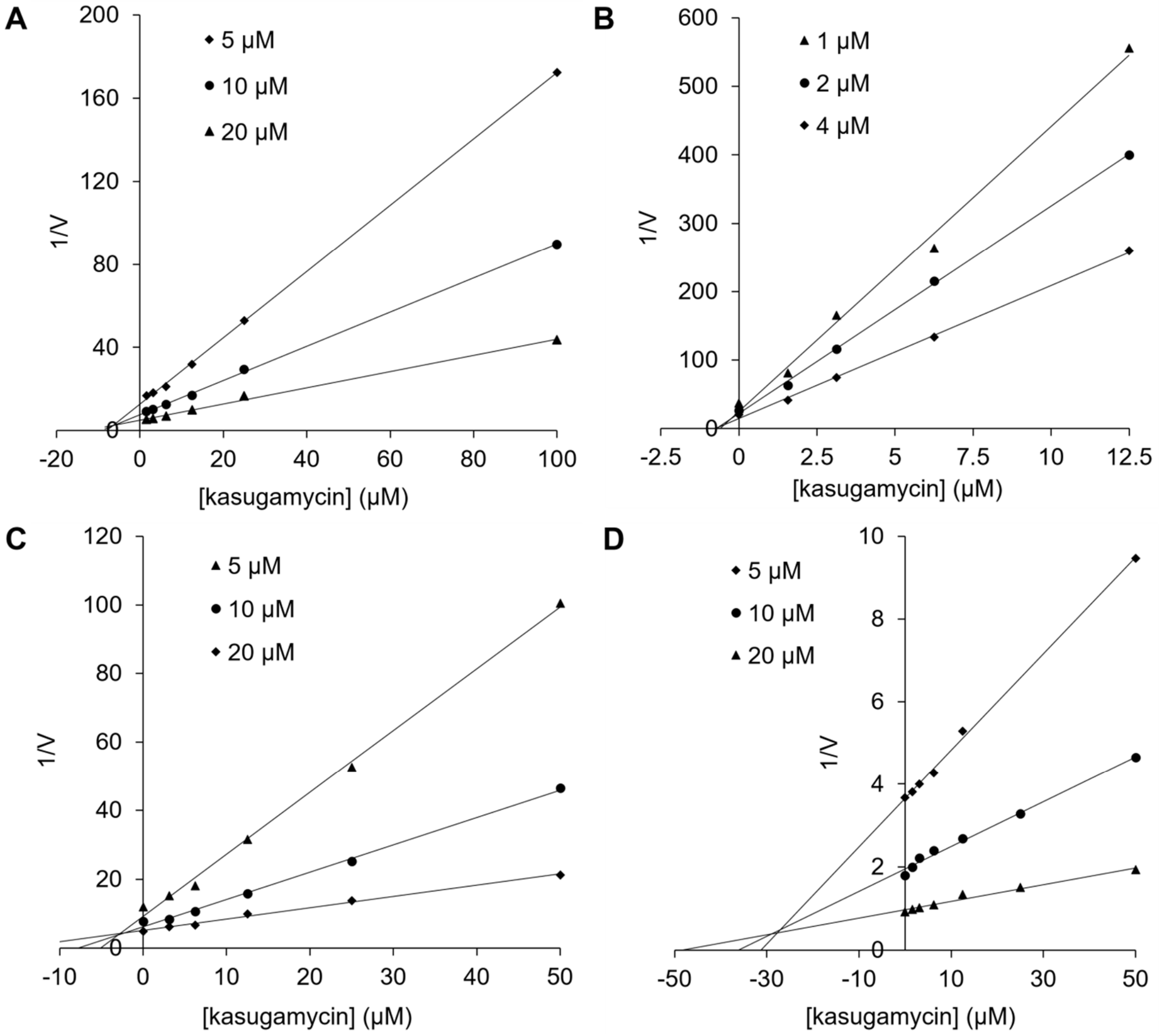

Figure S1. Inhibitory kinetics of kasugamycin against AMCase (A), *Of*ChtI (B), *Of*Chi-h (C) and *Sm*ChiA (D).

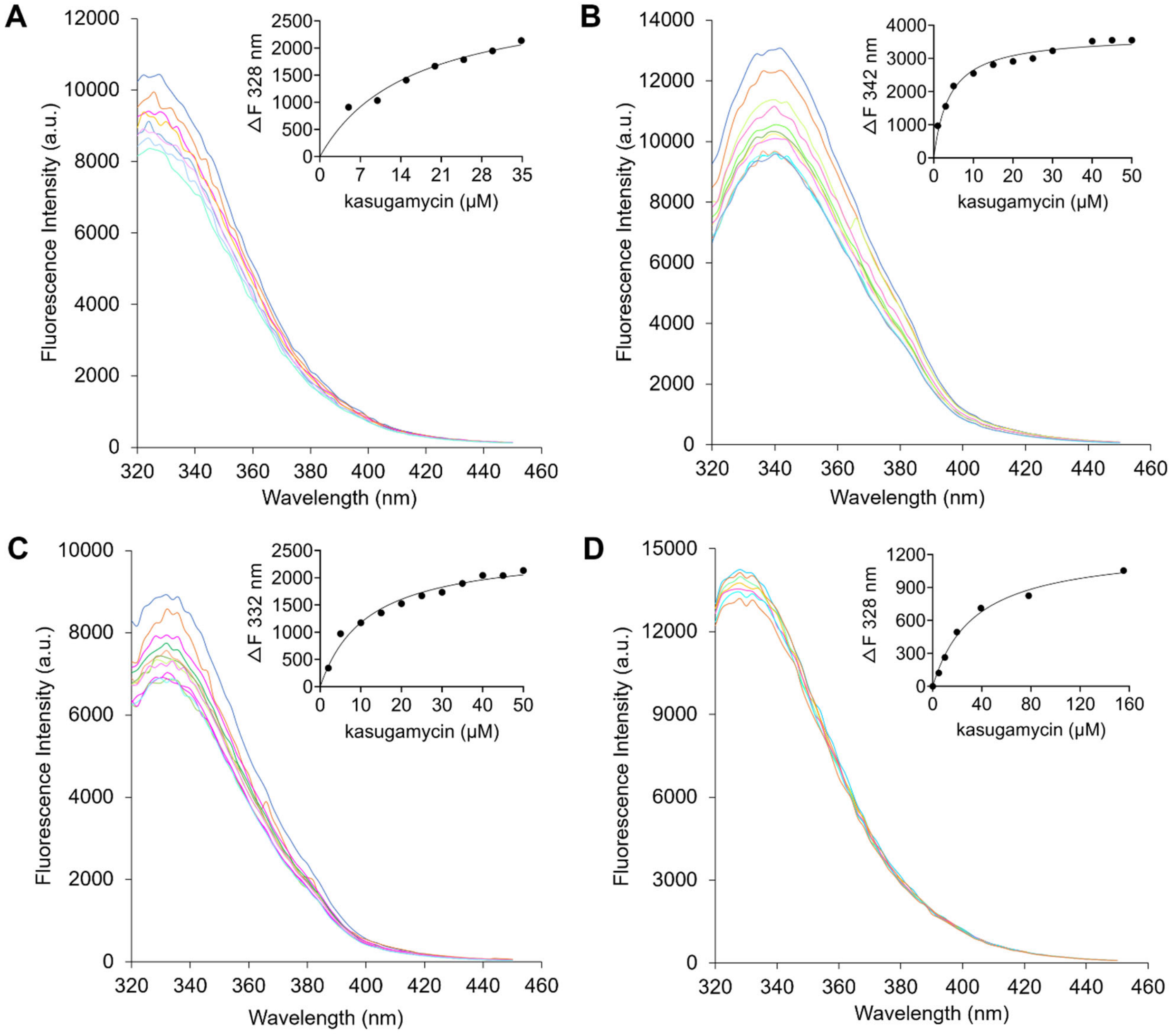

Figure S2. Binding affinities of kasugamycin to AMCase (A), *Of*ChtI (B), *Of*Chi-h (C) and *Sm*ChiA (D).

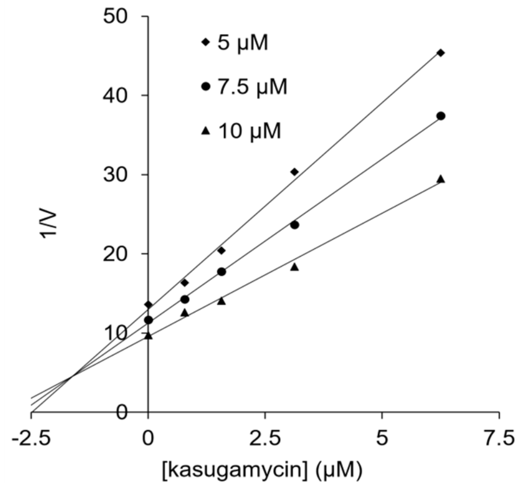

Figure S3. Inhibitory kinetic of kasugamycin against *Hs*Cht in buffer with 1.0 M NaCl.

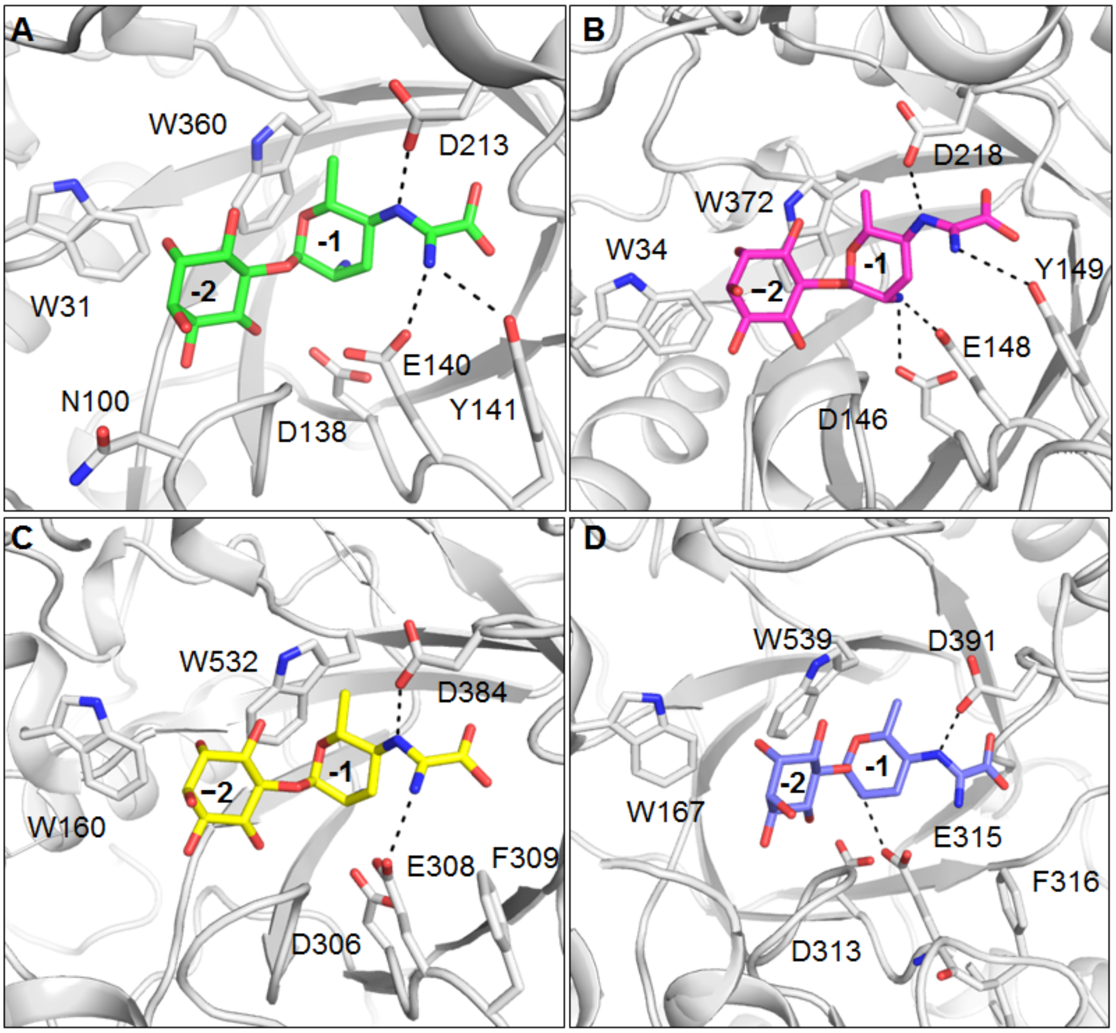

Figure S4. Modeled binding modes of kasugamycin to AMCase (A), *Of*ChtI (B), *Of*Chi-h (C) and *Sm*ChiA (D).
